# The Effect of Astaxanthin on Ochratoxin A-Induced Intestinal Injury in Chickens Through RIPK1/RIPK3/MLKL Pathway

**DOI:** 10.3390/antiox14080915

**Published:** 2025-07-25

**Authors:** Ruiwen Fan, Wenqi Tian, Bo Jin, Yuhang Sun, Miao Long, Shuhua Yang, Peng Li

**Affiliations:** College of Veterinary and Animal Science, Shenyang Agricultural University, Shenyang 110866, China; 15321759167@163.com (R.F.); syh2019@syau.edu.cn (Y.S.); longmiao@syau.edu.cn (M.L.)

**Keywords:** mycotoxins, ochratoxin A, astaxanthin, chickens, necroptosis, intestine

## Abstract

Ochratoxin A (OTA), as a mycotoxin, can contaminate a variety of feeds and foods. Existing studies have shown that the main toxicity of OTA to organisms is nephrotoxicity, but the toxic mechanism to other organs is still worthy of further study. Whether OTA causes intestinal damage through the necroptosis pathway mediated by *RIPK1/RIPK3/MLKL* remains to be elucidated. Astaxanthin (AST), a feed additive with strong antioxidant properties, was used as an antidote to evaluate the alleviation effect on OTA-induced intestinal injury and the underlying mechanism in this research. Chickens are the most sensitive animals to OTA except pigs. Therefore, 70 *white-feathered chickens* (*n* = 15) and Chicken Small Intestinal Epithelial Cells (CSIECs) were used as experimental subjects. Experimental models were established by single or combined exposure of OTA (1.0 mg/kg on chickens for 21 d; 2 μM on CSIEC for 24 h) and AST (100 mg/kg on chickens for 21 d; 40 μM on CSIEC for 24 h). In this study, AST significantly ameliorated OTA-induced intestinal damage by restoring the expression of tight junction proteins (*Occludin-1*, *Claudin-1*, and *ZO-1*), attenuating severe histopathological alterations, mitigating the inflammatory response (elevated pro-inflammatory cytokines and reduced anti-inflammatory mediators), and suppressing necroptosis through downregulation of *RIPK1*, *RIPK3* and *MLKL* expression. Combined evidence from animal experiments and cell culture experiments demonstrated that AST alleviated the necroptosis and inflammation caused by OTA in CSIECs and the intestine of chickens through the *RIPK1/RIPK3/MLKL* signaling pathway, thereby reducing the damage caused by OTA.

## 1. Introduction

Ochratoxin A (OTA), as an important mycotoxin, has attracted much attention since it was isolated and identified in 1965 [1,2]. There are three different subtypes of ochratoxins, namely OTA, ochratoxin B, and ochratoxin C, among which OTA has a particularly significant impact on public health [3,4,5]. OTA can be produced by several fungi of *Aspergillus* and *Penicillium*, and has been detected in the production of various agricultural products worldwide [6]. Animals that consume OTA-contaminated feed produce tissue bioaccumulation, and this toxin is detected in animal products such as milk and eggs [7,8,9]. Moreover, OTA has the characteristics of heat resistance, so it may persist under food processing conditions of 80–121 °C [10,11]. OTA can lead to reduced feed efficiency, liver and kidney damage, immunosuppression, and teratogenicity in animals, especially pigs and poultry, and has been classified as a possible carcinogen by the International Agency for Research on Cancer (IARC) [12,13]. The European Commission limits the maximum limit of OTA in feed ingredients represented by cereals and cereal products to 250 μg/kg, and the maximum limit of poultry supplementary feed and compound feed is 100 μg/kg [14]. The intestine is one of the important target organs of OTA, and oxidative stress is one of the most important mechanisms of its toxic effects. Wang et al. found that OTA (10–40 μM, 12 h) exposure increased ROS production and induced intestinal oxidative stress in IPEC-J2, eventually leading to intestinal barrier dysfunction and tight junction destruction [15]. Even long-term low-dose OTA (0.05 mg/kg, 30 d) exposure on *crossbred weaned piglets (TOPIGS-40)* affected the immune response at the gut, and the gene expression of inflammation signaling pathways and inflammatory cytokines in the gut were higher than in the kidneys [16]. In addition, OTA (300–500 μg/kg, 28 d) exposure has been shown to induce apoptosis of the intestinal epithelium in *Wistar rats* and make the body more vulnerable to harmful substances and pathogens [17,18]. The structure of intestinal microbial flora is also crucial for the operation of intestinal function. The research of Xia et al. found that OTA (500 μg/kg, 28 d) induced *Cherry Valley ducklings*’ inflammatory response through *TLR4* signaling pathway, increased the relative abundance of *Bacteroidetes*, and decreased the relative abundance of *Firmicutes* in the cecum, resulting in the accumulation of LPS [19].

Necrosis, due to its lack of a fixed form (except for early plasma membrane rupture), is traditionally regarded as a non-regulatory death caused by nonspecific stress [20]. Recent studies have shown that necrosis can also be regulated by specific mechanisms, especially in the case of inhibition of *caspase-8*. It can also be regulated by death domain receptor-related kinases known as necroptosis [21]. Hitomi et al. revealed a gene network that mediates necroptosis [22]. The key step in activating necroptosis is the activation of *RIPK1* kinase [23]. Phosphorylation of *RIPK1* at the appropriate serine residue activates *RIPK3* and *MLKL* kinases to form complex IIb under *caspase-8* inhibition, leading to necroptosis [24]. After phosphorylation of *RIPK3*, *MLKL* will form oligomers that promote ion inflow (calcium and sodium) or pore formation and transfer to the plasma membrane, which is the cause of a loss of membrane integrity. This membrane rupture causes inflammation, which ultimately leads to cell death by releasing cell contents [25]. Studies have shown that OTA induces necroptosis in the liver and intestine of *mice* (0.5–8 mg/kg, 7 d) and *grass carp* (1.2 mg/kg, 60 d) through the *RIP/MLKL* signaling pathway [26,27]. As a global pollution mycotoxin, OTA seriously threatens the health and food safety of livestock and poultry due to its strong toxicity, bioaccumulation, and thermal stability. However, at present, the main research focus of OTA-induced injury is still liver and kidney oxidative stress [28]. Therefore, it is quite important to find latent toxic effects of OTA-induced body damage and find green pharmaceuticals for prevention or treatment. Chickens are highly adaptable to toxicants due to their elevated metabolic rate and extended lifespan [29]. Chickens are the most sensitive animals to OTA except pigs; therefore, this study selected chickens as research animals. According to Han’s research, after application of OTA (0.5, 1.0, 2.0 mg/kg, 7 d) to *white-feathered broilers* (*n* = 7), 1 and 2 mg/kg OTA extremely significantly reduced the content of GSH-Px and SOD, but in the 2 mg/kg group two broilers (29%) died, which indicated the concentration of 2 mg/kg OTA was too high and the toxicity was too strong [30]. Therefore, in this experiment, 1 mg/kg OTA concentration was applied to *white-feathered broilers* for 21 days to observe its toxicity on the intestine. Based on the well-documented intestinal toxicity of OTA, this study employed Chicken Small Intestinal Epithelial Cells (CSIECs), a well-established in vitro model for poultry intestinal research, to investigate OTA-induced toxic mechanisms in avian intestinal epithelium at the cellular level.

Astaxanthin (AST), a member of the xanthophyll family, is recognized as a safe and potent natural antioxidant [31]. Studies have found that AST has a variety of biological effects (strong antioxidant effect, resistance to inflammation, and other protective effects) [32]. The main reason why AST has attracted widespread attention is its strong antioxidant activity; it is more active than common antioxidants such as lutein, vitamins (C, E), and β-carotene [33]. More and more studies have shown that AST could also play a therapeutic role through its anti-apoptotic properties [34]. It has been confirmed that AST could activate *PI3K/Akt* pathway, downregulate the ratio of *Bax/Bcl-2*, and inhibit the activity of *caspase-3/9* to resist apoptosis [35,36]. In a respiratory system injury model, AST showed significant anti-inflammatory lung injury protection by regulating the activity of inducible nitric oxide synthase (*iNOS*), inhibiting the production of nitrotyrosine (NT), blocking activation of *NFκB P65* subunit, and inhibiting the programmed cell death pathway of lung tissue [37]. As a natural strong antioxidant, AST has the biological characteristics of anti-inflammation, anti-apoptosis, and regulation of cell signaling pathways, which has become an ideal candidate of this research. However, the molecular mechanism of whether AST interferes with OTA-induced intestinal injury by regulating the necroptosis pathway is not clear. According to Zou’s research, 100 mg/kg AST effectively alleviated the oxidative stress and endoplasmic reticulum stress caused by 1.0 mg/kg OTA in *white-feathered broilers*, and restored the morphology and function of the endoplasmic reticulum and mitochondria [38]. Therefore, 100 mg/kg AST was selected as a protective drug to alleviate 1.0 mg/kg OTA poisoning on intestine of chickens in this research.

Therefore, in this study, the toxic effects of OTA on the intestine of chickens and the intervention mechanism of AST were systematically elucidated by combining animal experiments and cell culture experiments. The purpose of this study was to reveal the mechanism of AST alleviating OTA-induced intestinal injury in chickens through *RIPK1/RIPK3/MLKL* necroptosis signaling pathway. The research seeks to clarify the role of AST in inhibiting intestinal epithelial cell necroptosis and restoring barrier function, thereby filling the knowledge gap regarding protective mechanisms of AST against OTA toxicity.

## 2. Materials and Methods

### 2.1. Chemicals and Reagents

AST for chickens was supplemented in the form of *Haematococcus pluvialis* powder (Ruihai Jinke Biotechnology Co., Ltd., Shanghai, China), and the effective content of AST is 3% (*Haematococcus pluvialis* powder is a natural product extracted from freshwater microalgae *Haematococcus pluvialis* and is one of the most important commercial sources of natural AST). *Aspergillus ochraceus* AS3.3876 was provided by Guangdong Microbiological Culture Collection Center (Guangzhou, China). NO content determination reagent was provided by Nanjing Jiancheng Bioengineering Institute (A012-1-2, Nanjing, China). ELISA kits of interleukin (IL-1β, ELK1979, CSIEC; IL-6, ELK1607, tissue homogenates of chicken) were bought from ELK Biotechnology Co., Ltd. (Wuhan, China). ELISA kits of IL-1β (YPJ1888, tissue homogenates of chicken) were bought from UpingBio Co., Ltd. (Shenzhen, China).

The pure OTA toxin was purchased from Prubang Bioengineering Co., Ltd. (HPLC ≥ 98%, Qingdao, China). AST for CSIEC (HPLC ≥ 98%) was purchased from Yuanye Biotechnology Co., Ltd. (Shanghai, China). Necrostatin-1 (Nec-1) was purchased from MedChemExpress (HY-15760, Monmouth Junction, NJ, USA) and was dissolved in dimethyl sulfoxide (DMSO) (D8370, Solarbio, Beijing, China).

All primary antibodies for Western Blot were purchased from ImmunoWay (Plano, TX, USA): RIPK1 rabbit mAb (YN1850, at 1:1000 dilution), RIPK3 rabbit mAb (YN1882, at 1:1000 dilution), MLKL rabbit mAb (YM8455, at 1:1000 dilution), Occludin rabbit mAb (YN2865, at 1:1000 dilution), Claudin-1 rabbit mAb (YT0942, at 1:1000 dilution), and β-actin rabbit mAb (YM8010, at 1:1000 dilution). HRP-labeled goat anti-rabbit IgG antibody was selected as the secondary antibody (RS0011, at 1:1000 dilution) in this research, which was purchased from ImmunoWay (Plano, TX, USA).

### 2.2. Preparation of OTA-Contaminated Moldy Feed

#### 2.2.1. Activation and Culture of Bacteria

The strain of *Aspergillus ochraceus* AS3.3876 was spread on LB solid medium (Solarbio Science & Technology Co., Ltd., Beijing, China) and cultured in an incubator at 28 °C. The colonies with good growth status were picked and inoculated in LB liquid medium after being autoclaved, and were cultured in a constant-temperature shaker at 28 °C until white round pellets with uniform size and in good condition grew in a conical flask.

#### 2.2.2. Preparation of Moldy Feed

The LB liquid medium containing *A. ochraceus* in good growth condition was evenly sprayed into the feed twice a day. The inoculated feed was incubated in a constant-temperature incubator at 28 °C for 14 days. After that, moldy feed was collected and autoclaved.

#### 2.2.3. Determination of OTA Concentration in Moldy Feed

The 5 g feed sample and 1 g NaCl were accurately transferred to a centrifuge tube and dissolved in a 20 mL methanol–water system (volume ratio 4:1). Ultrasonic treatment for 30 min promoted the dissolution of active ingredients. After filtration by organic-phase filter membrane, 1 mL filtrate was mixed with 200 μL chloroform and transferred to a centrifuge tube containing 5 mL acetic acid solution. The upper aqueous phase was discarded, and the organic phase was retained. The residue was dried by mild nitrogen, dissolved in 100 μL of chromatographic-grade methanol, filtered by organic-phase filter membrane, and used for concentration determination by liquid chromatography.

Following successful preparation of mold-contaminated feed, the OTA concentration was quantified by high-performance liquid chromatography with ultraviolet detection (HPLC-UV, Thermo Fisher Scientific, Waltham, MA, USA). A standard curve was established using serial dilutions of OTA reference standards, yielding the linear regression equation y = 24.3520x − 8.9629 (R^2^ = 0.99985). The chromatographic peak area of OTA in the contaminated feed sample was measured as 223.180. Based on the standard curve, the OTA concentration in the moldy feed was determined to be 38.1312 μg/kg.

### 2.3. Animal Research

All animal experimental procedures were conducted in compliance with the ethical guidelines approved by the Shenyang Agricultural University Institutional Animal Care and Use Committee (approval number: 201806014). In order to reduce the interference of potential pathogenic microorganisms, a multi-tiered brooding cage system was used throughout the experiment. The ambient temperature was constant at 32 °C (fluctuation range ±5 °C), and the relative humidity was controlled at 40 ± 5%. All individuals were free to obtain standardized diets and sterile drinking water, implement standardized environmental disinfection procedures every day, and maintain air quality through mechanical ventilation systems to ensure that experimental conditions were in line with animal welfare norms.

In this experiment, 70 1-day-old *white-feathered chickens (Ross 308)* were selected as experimental animals, which were purchased from Harbin Weiwei company (Harbin, China). After one week of adaptive feeding, the individuals with stress response and developmental delay were excluded, and 60 individuals were finally determined to reach the standard. The 60 chickens were randomly divided into 4 groups (*n* = 15): control group (normal drinking water and basic feed), OTA group (moldy feed and normal feed were evenly mixed in a certain proportion to an OTA concentration of 1.0 mg/kg, the feed was freshly prepared and administered daily, normal drinking water), AST group (normal feed and *Haematococcus pluvialis* powder were evenly mixed in proportion to ensure that the AST concentration in the feed was 100 mg/kg, normal drinking water), and OTA + AST group (OTA concentration in the feed was 1.0 mg/kg, AST concentration was 100 mg/kg, normal drinking water). The body weight of chickens was recorded daily, and the chickens were sacrificed on the 21st day of the experiment. Whole blood samples were collected and serum was separated, and the jejunum tissue was completely intercepted for subsequent experiments.

### 2.4. HE Staining

The histomorphological analysis of this study was commissioned by Servicebio Technology Co., Ltd. (Hubei, China), with pathological diagnosis qualification, including paraffin-embedded section preparation and hematoxylin–eosin staining. The sections were examined and photographed by optical microscope. The villus length and crypt depth, and the ratio of the two, were measured by using ImageJ 1.54g software.

### 2.5. NO Detection

The detection process strictly followed standardized operation specifications provided by the following manufacturer, and NO of the jejunum was quantitatively analyzed by colorimetry. All the color reactions were detected at a wavelength of 550 nm by a microplate reader (Tecan Trading Co., Ltd., Männedorf, Switzerland).

### 2.6. CCK8 to Determine Cell Viability and Cell Culture with Treatment

CSIECs (Pricella Biotechnology Co., Ltd., Wuhan, China) were cultured in DMEM/F12 medium. A total of 50 mL of 10% fetal bovine serum (Pricella, Wuhan, China) and 1% penicillin–streptomycin double-antibody mixture (Solarbio, Beijing, China) were added and cultured in a humidified incubator at 37 °C and 5% CO_2_.

For cell viability assay, CSIECs were seeded in a 96-well plate at a density of 1 × 10^4^ cells per well and incubated in an incubator at 37 °C and 5% CO_2_ for 24 h. When the cells reached 60–70% confluence, different concentrations of OTA treatment groups (1 μM, 2 μM, 3 μM, 4 μM, 5 μM) and AST treatment groups (5 μM, 10 μM, 20 μM, 40 μM, 80 μM, 100 μM) were prepared, and a combined treatment group containing both substances was set. The old medium was removed, 100 μL of the configured OTA or AST solution was added to each well, 6 replicates were set for each concentration, and they were treated for 24 h. After removing the treatment medium, a medium containing 10% CCK8 solution (Glpbio, Montclair, NJ, USA) was added and incubated for 1 h. The absorbance value (OD value) of each hole at 450 nm wavelength was measured by microplate reader. The cell viability of the CON group was set to 100% as a benchmark, and the relative cell viability of each experimental group was calculated according to the formula below: cell viability(%) = [(experimental group OD value − blank group OD value)/(control group OD value − blank group OD value)] × 100%.

In order to establish an OTA-induced necroptosis model in CSIECs, we conducted two in vitro experiments according to the results of CCK8. (1) AST antagonistic experiment: The cells were divided into four groups: control group (only DMEM/F12 basal medium was added), OTA group (2 μM OTA), AST group (40 μM AST), and OTA + AST group (40 μM AST + 2 μM OTA). (2) RIP1 inhibitor Nec-1 intervention experiment: The cells were divided into three groups: control group (only DMEM/F12 basic medium was added), AST + OTA group (40 μM AST + 2 μM OTA), OTA + AST + Nec-1 group (40 μM AST + 2 μM OTA + 3 μM Nec-1), and treated for 24 h.

### 2.7. Flow Cytometry

CSIECs in good growth condition were selected and added to a 6-well plate, and the culture plate was treated as mentioned above. Three repeated samples were set for each condition. According to the manufacturer’s instructions and different detection requirements, Annexin V Apoptosis Detection Kit (E-CK-A211, Elabscience Biotechnology Co., Ltd., Wuhan, China) was used to stain the cell suspension. The cells were gently mixed and placed in a dark environment at room temperature for 20 min. Finally, data were analyzed by flow cytometry (Beckman Coulter GmbH, Krefeld, Germany).

### 2.8. Analysis of Interleukin Index

The levels of interleukin (IL-1β, IL-6) in tissues and cells were both measured by ELISA kits, and the operation steps were carried out according to the instructions. At least 6 replicates were performed on each set of data. The level of IL-1β and IL-6 in each sample was calculated according to the protocol of the manufacturer.

### 2.9. Detection of Related Gene Expression

Samples were lysed by an RNA isolator (R401-01, Vazyme Biotech Co., Ltd., Nanjing, China). The RNA of chicken intestine and CSIECs was purified step by step by chloroform, isopropanol, and 75% ethanol. The ratio of OD260/OD280 in this research was 1.8–2.1, which indicated that the RNA of samples met the RNA purity requirement. The RNA was reverse transcribed into cDNA by using a reverse transcription kit (R323-01, Vazyme, Nanjing, China). SYBR green real-time fluorescence quantitative PCR was used for quantitative analysis. The information of all specific primer sequences (HPLC ≥ 98%) is shown in Table 1 below. The volume of the qRT-PCR system was 20 µL, which included the SYBRGREEN mix (10 μL, Q711-02, Vazyme, Nanjing, China), upstream primer (10 μmol/L, 0.4 μL), downstream primer (10 μmol/L, 0.4 μL), sample 2.0 μL, and ddH_2_O 6.2 μL. Finally, mRNA expression level was calculated by the 2^−△△Ct^ method.

### 2.10. Western Blotting of Protein Expression

Phosphatase inhibitors and protease inhibitors were added to the RIPA lysate, and the above mixture (Yamei Biotechnology Co., Ltd., Shanghai, China) was added to tissues and cells to extract total protein. Then, the concentration of total protein was measured by using a BCA kit (A045-4-2, Nanjing Jiancheng, Nanjing, China). Loading buffer and ddH_2_O were added to the sample after calculation.

A total of 20 μL protein sample was separated by electrophoresis using 7–15% SDS-PAGE gel (Yamei, Shanghai, China). Subsequently, the protein was transferred to the PVDF membrane. The membrane was sealed in fast sealing solution (Yamei, Shanghai, China) for 1.5 h and then was incubated overnight in primary antibody at 4 °C. A solution of 1 × TBST (diluted from 10 × TBST solution, Servicebio, Wuhan, China) was used to wash the membrane 6 times, 5 min each time, the next day. Then, the membrane was incubated with secondary antibody for 1.5 h at room temperature, and the above washing operation was repeated. After the result was obtained, the gray value of the band was analyzed by ImageJ software.

### 2.11. Statistical Analysis

The statistical analyses were conducted by using IBM SPSS Statistics 25 software (Chicago, IL, USA). The results were expressed as mean ± standard deviation (Mean ± SD). For group comparisons, one-way analysis of variance followed by Tukey multiple comparisons test were performed. Graphical representations were generated using GraphPad Prism 9 (La Jolla, CA, USA). *p* < 0.05 indicated significant difference, while *p* < 0.01 indicated extremely significant difference.

## 3. Results

### 3.1. AST Alleviated the Damage of OTA on Chickens and CSIECs

**Figure 1:**
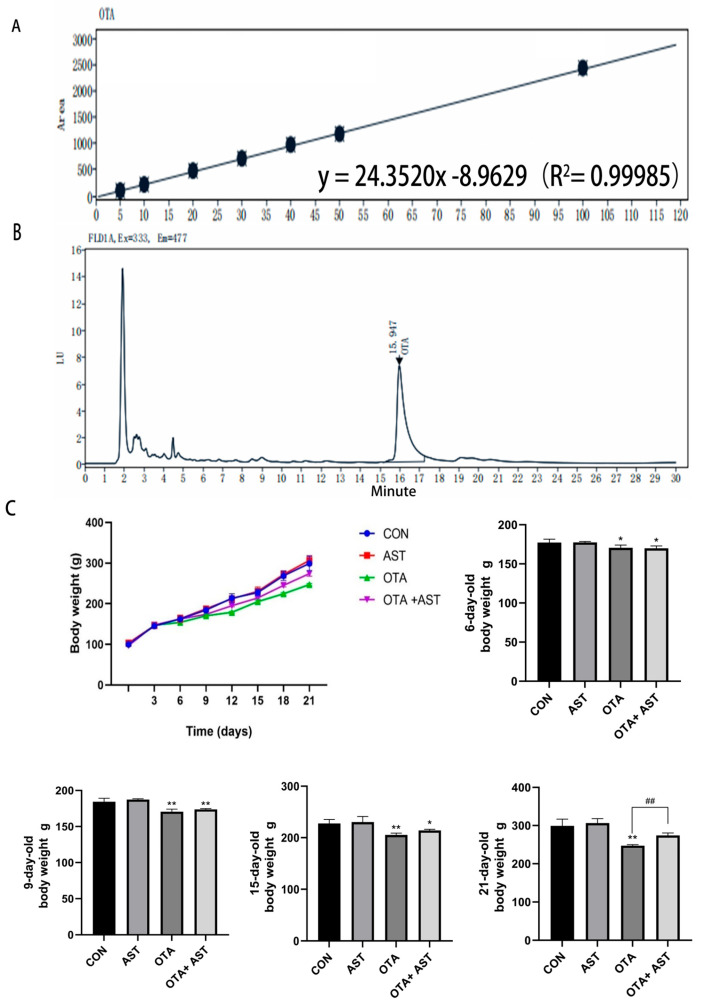
AST alleviated the damage of OTA on chickens and CSIECs. (**A**) Standard curve of OTA standard. (**B**) OTA sample chromatogram. (**C**) Chicken growth curve and chicken weight changes (*n* = 6). Values are expressed as AVG ± SD. * *p* < 0.05 vs. control; ** *p* < 0.01 vs. control; ## *p* < 0.01 vs. OTA group.

It can be seen from Figure 1A,B that the moldy feed was successfully constructed. In order to explore whether AST can alleviate the damage of OTA to chickens, we recorded the changes in body weight. Compared with the CON group, the body weight of chickens in the OTA group and OTA + AST group decreased to varying degrees. From the sixth day, the addition of OTA could make the body weight of chickens significantly lower than in the CON group (*p* < 0.05), indicating that OTA played a negative regulatory role in the growth of chickens. At 21 days of age, the body weight of chickens in the OTA + AST group was significantly higher than in OTA group (*p* < 0.01) (Figure 1C).

### 3.2. AST Alleviated the Inflammatory Damage Caused by OTA to the Jejunum of Chickens

**Figure 2:**
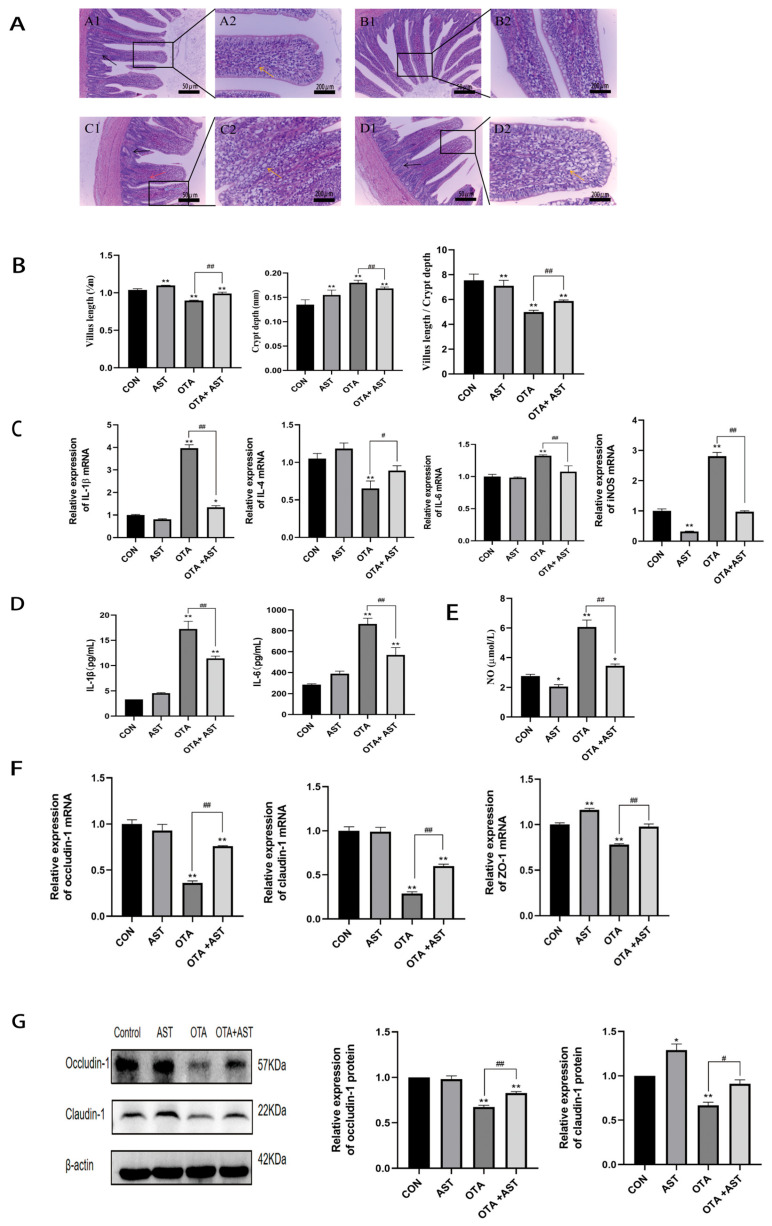
AST alleviated the inflammatory damage caused by OTA to the jejunum of chickens. (**A**) HE staining results for jejunum tissues of chickens. (**A1**,**B1**,**C1**,**D1**) Ileal structure in 100-fold visual field, (**A2**,**B2**,**C2**,**D2**) Ileal structure in 400-fold visual field. (**A1**,**A2**) Control group, (**B1**,**B2**) AST group, (**C1**,**C2**) OTA group, (**D1**,**D2**) OTA + AST group. Black arrow: crypts. Red arrow: bleeding phenomenon. Blue arrow: inflammatory cell infiltration. Yellow arrow: goblet cells. (**B**) Results of AST and OTA on ileal morphology of chickens. (**C**) Results of OTA and AST on the mRNA content of *iNOS and IL-1β, IL-4, IL-6* in chickens (*n* = 6). (**D**) Results of OTA and AST on the content of IL-1β, IL-6 in chickens (*n* = 6). (**E**) Results of OTA and AST on the content of NO in chickens (*n* = 6). (**F**) Results of OTA and AST on the mRNA of tight junction proteins in chickens (*n* = 6). (**G**) Results of OTA and AST on the relative content of tight junction proteins in chickens (*n* = 3). Values are expressed as AVG ± SD. * *p* < 0.05 and ** *p* < 0.01 vs. control group; # *p* < 0.05 and ## *p* < 0.01 vs. OTA group.

We evaluated the influence of OTA and AST on intestinal structure by conducting HE staining on jejunum tissues of chickens. As shown in Figure 2A, the OTA group showed inflammatory cell infiltration, irregular arrangement of crypts and villi, structural changes of crypts, a reduction in goblet cells, and bleeding compared with the CON group; compared with the OTA group, the inflammatory cell infiltration in the OTA + AST group was reduced, the crypts and villus morphology were arranged regularly, the structure was complete, and the goblet cells increased. It is suggested that OTA can lead to the destruction of the intestinal tissue structure of chickens, and AST can effectively alleviate this phenomenon. We further found that, as shown in Figure 2B, the villus length of the OTA group was significantly shortened (*p* < 0.01), the crypt depth was significantly increased (*p* < 0.01), and the ratio of these two was significantly decreased (*p* < 0.01) compared with the CON group; after AST treatment, the above changes were significantly alleviated (*p* < 0.01). These results show that OTA could cause damage to the jejunum villi and crypts of chickens, but AST can reduce the damage to the ileum.

Intestinal damage increases permeability, which allows pathogens or external stimuli to trigger tissue inflammation. As shown in Figure 2C,D, mRNA expression of *IL-1β*, *IL-6*, and *iNOS*, and the concentration of IL-1β and IL-6 in the serum of chickens in the OTA group, were significantly increased (*p* < 0.01), while the mRNA expression of *IL-4* and the concentration in the serum were significantly decreased (*p* < 0.01) compared with the CON group. Compared with the OTA group, the above indicators showed significant to extremely significant recovery after the addition of AST. During intestinal inflammation, altered *iNOS* activity leads to increased NO production. The NO concentration in the serum of chickens in the OTA group was significantly increased (*p* < 0.01) compared with CON group. However, the serum NO concentration in the OTA + AST group was significantly restored (*p* < 0.01) compared with the OTA group (Figure 2E). This indicates that OTA caused severe inflammatory reaction in the intestine of chickens, and the inflammation was alleviated to a large extent after AST treatment.

Excessive production of NO will affect signal transduction and lead to abnormal intestinal barrier function. In this study, the effects of OTA on intestinal barrier function of chickens and the intervention effect of AST were evaluated by detecting the gene and protein expression levels of key tight junction proteins *(Occludin-1, Claudin-1 and ZO-1)* of the intestinal mucosal barrier. The results show that the expression levels of tight junction proteins in the OTA exposure group were significantly lower than those in the CON group at the mRNA level (*p* < 0.01); the OTA + AST group was significantly upregulated compared with the OTA exposure group (*p* < 0.01) (Figure 2F). At the protein level, the expression changes in *Occludin-1* and *Claudin-1* were consistent with the mRNA level, and AST intervention completely restored the expression of *Claudin-1* protein inhibited by OTA to a normal level (Figure 2G). These results indicate that OTA can significantly damage the intestinal barrier function of chickens, and AST can effectively alleviate this damage.

The results show that OTA induced intestinal inflammation in chickens, thereby damaging intestinal structure and barrier function, and these negative effects were significantly alleviated after AST intervention.

### 3.3. AST Alleviated OTA-Induced Necroptosis in the Chicken Jejunum by Modulating the RIPK1/RIPK3/MLKL Signaling Pathway

**Figure 3:**
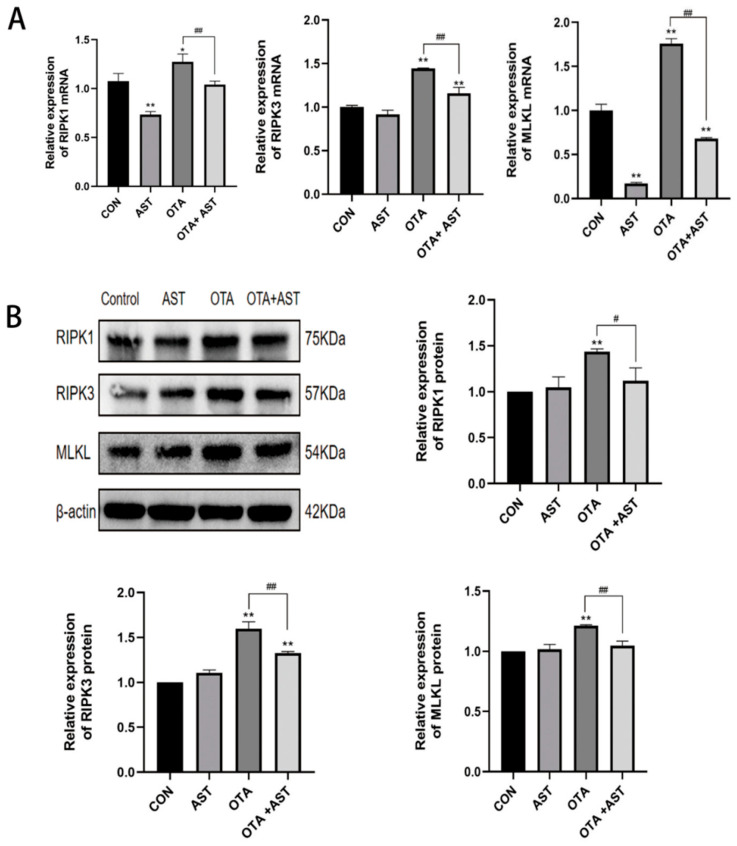
AST alleviated OTA-induced necroptosis in the chicken jejunum by modulating the *RIPK1/RIPK3/MLKL* signaling pathway. (**A**) Results of OTA and AST on the mRNA content of *RIPK1/RIPK3/MLKL* pathway in chickens (*n* = 6). (**B**) Results of OTA and AST on key protein content of *RIPK1/RIPK3/MLKL* pathway in chickens (*n* = 3). Values are expressed as AVG ± SD. * *p* < 0.05 and ** *p* < 0.01 vs. control group; # *p* < 0.05 and ## *p* < 0.01 vs. OTA group.

The purpose of this research was to further analyze the mechanism of OTA-induced necroptosis in the intestine, so the regulation of *RIPK1/RIPK3/MLKL* pathway was focused on. The results of qRT-PCR show that OTA exposure could significantly upregulate the mRNA expression levels of *RIPK1*, *RIPK3*, and *MLKL* genes in intestinal tissues of chickens (*p* < 0.01), while AST treatment could effectively reverse this gene expression change (Figure 3A). The results of protein level detection were consistent with the trend of mRNA expression, and Western blot analysis further confirmed the above findings (Figure 3B). It indicated that AST alleviated the necroptosis of OTA on chicken jejunum by interfering with *RIPK1/RIPK3/MLKL* signaling pathway.

### 3.4. AST Alleviated the Inflammatory Damage Caused by OTA on CSIECs

**Figure 4:**
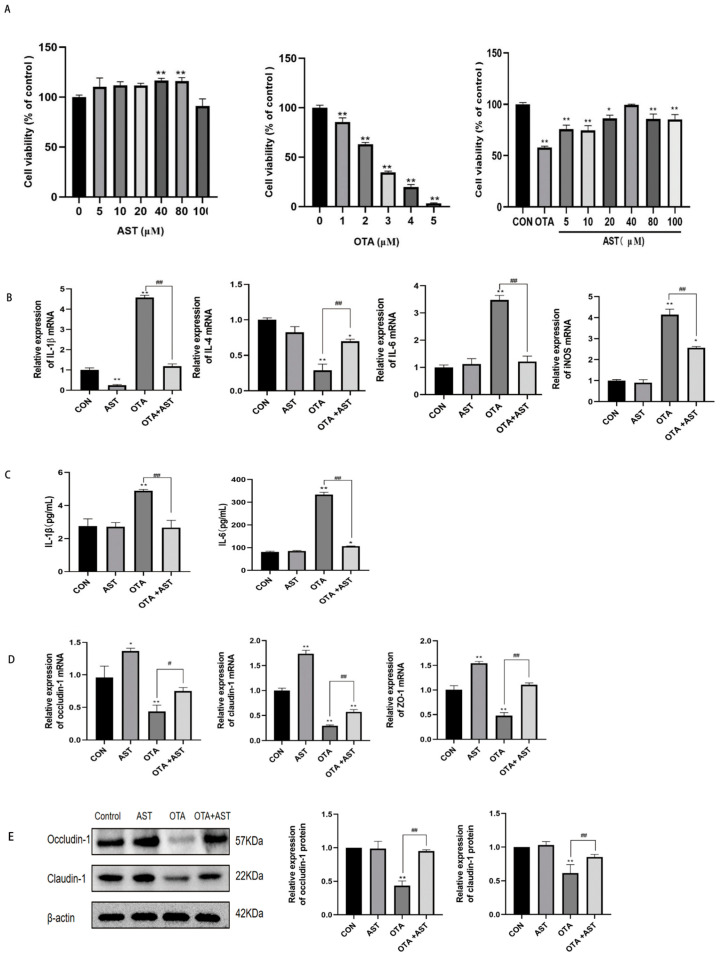
AST alleviated the inflammatory damage caused by OTA on CSIECs. (**A**) Results of AST and/or OTA on cell viability (*n* = 6). (**B**) Results of OTA and AST on the mRNA content of *IL-1β*, *IL-4*, *IL-6*, and *iNOS* in the supernatant of CSIECs (*n* = 6). (**C**) Results of OTA and AST on the content of IL-1β and IL-6 in CSIEC (*n* = 6). (**D**) Results of OTA and AST on the mRNA of tight junction proteins in CSIECs (*n* = 6). (**E**) Results of OTA and AST on the relative content of tight junction proteins in CSIECs (*n* = 3). Values are expressed as AVG ± SD. * *p* < 0.05 and ** *p* < 0.01 vs. control group; # *p* < 0.05 and ## *p* < 0.01 vs. OTA group.

In determining the appropriate concentration of the two drugs for CSIECs, CCK8 results show that AST (0–100 μM) was non-toxic to CSIECs; compared with the CON group, the cell viability of CSIECs in OTA group decreased in a dose-dependent manner with the increase in OTA concentration, the cell viability of CSIECs decreased significantly from 1 μM OTA (*p* < 0.01), and the cell viability was 62% at 2 μM OTA. Therefore, 2 μM OTA with a cell viability of about 60% was selected for subsequent experiments; compared with the OTA group, the combined group had the best effect at AST 40 μM, and there was no significant difference in cell viability compared with the CON group. Therefore, 2 μM OTA and 40 μM AST were selected for subsequent experiments (Figure 4A). The above results indicate that OTA can cause damage to chickens and CSIECs, and AST can alleviate its side effects.

In the process of intestinal inflammation, the role of inflammatory factors will lead to the expression of *iNOS*. At the same time, in order to further explore the inflammation of OTA combined with AST on CSIECs, we detected the mRNA expression of *IL-1β*, *IL-4*, *IL-6*, and *iNOS* in CSIECs. Compared with the control group, the expression of pro-inflammatory factors *IL-1β*, *IL-6*, and *iNOS* mRNA in the OTA group was significantly increased (*p* < 0.01), and the expression of anti-inflammatory factor *IL-4* mRNA was significantly decreased (*p* < 0.01). Compared with the OTA group, the expression of *IL-1β*, *IL-6*, and *iNOS* mRNA in the OTA + AST group was significantly decreased (*p* < 0.01), and the expression of *IL-4* mRNA was significantly increased (*p* < 0.01) (Figure 4B). Similar trends were also observed in the detection of IL-1β and IL-6 inflammatory factor concentrations in CSIEC supernatants using ELISA kits (Figure 4C).

Excessive iNOS can lead to intestinal barrier dysfunction, so we evaluated the protein and mRNA expression of key tight junction proteins in CSIECs, which are crucial for maintaining the intestinal mucosal barrier. As illustrated in Figure 4D,E, OTA exposure significantly reduced both gene and protein expression of these tight junction proteins compared to the control group (*p* < 0.01). However, co-treatment with AST effectively counteracted the OTA-induced suppression of tight junction proteins. It indicated that OTA damaged the ileal barrier of chickens, and the damages were alleviated after AST treatment.

The above results show that OTA caused CSIEC inflammation and further damaged the intestinal barrier index, but AST could reduce these damages.

### 3.5. AST Alleviated the Necroptosis of OTA on CSIECs by Interfering with RIPK1/RIPK3/MLKL Pathway

**Figure 5:**
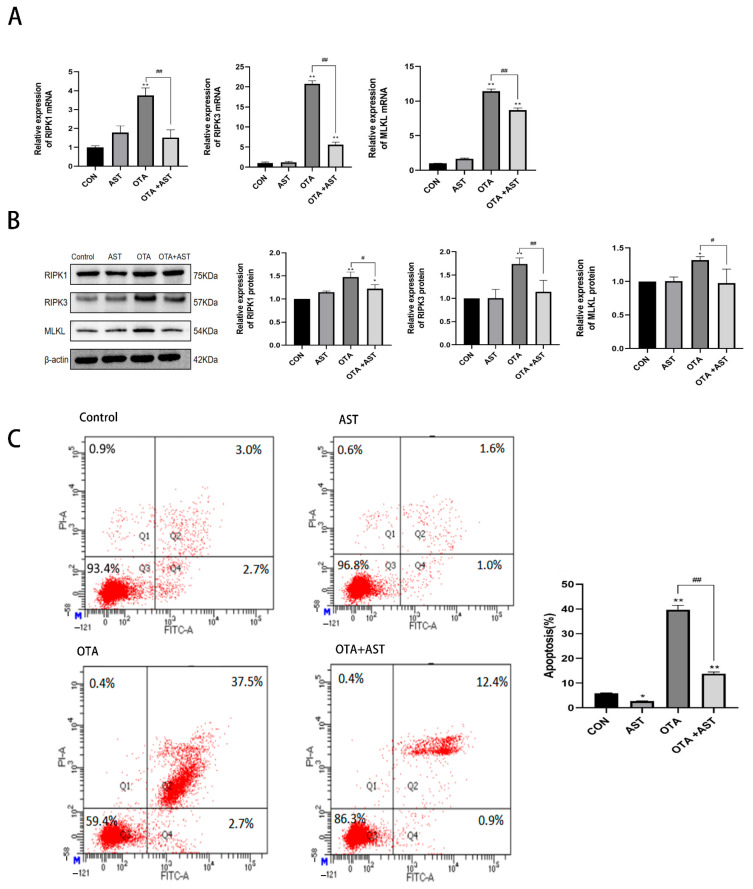
AST alleviated the necroptosis of OTA on CSIECs and chicken jejunum by interfering with *RIPK1/RIPK3/MLKL* signaling pathway. (**A**) Results of OTA and AST on the mRNA content of *RIPK1/RIPK3/MLKL* pathway in CSIECs (*n* = 6). (**B**) Results of OTA and AST on key protein content of *RIPK1/RIPK3/MLKL* pathway in CSIECs (*n* = 3). (**C**) Results of OTA and AST on necroptosis of CSIECs detected by flow cytometry (*n* = 3). Values are expressed as AVG ± SD. * *p* < 0.05 and ** *p* < 0.01 vs. control group; # *p* < 0.05 and ## *p* < 0.01 vs. OTA group.

In order to explore the mechanism of AST alleviating OTA-induced intestinal necroptosis in the cell experiment, we evaluated the regulatory effect of *RIPK1/RIPK3/MLKL* signaling pathway in CSIECs. The results of qRT-PCR showed that OTA treatment could significantly increase the mRNA expression levels of *RIPK1*, *RIPK3*, and *MLKL* genes in CSIECs (*p* < 0.01), and AST intervention could effectively inhibit the expression changes of these genes induced by OTA (Figure 5A). At the protein level, the results of Western blot analysis were consistent with the trend of mRNA expression, which further verified the above findings (Figure 5B). In order to verify whether AST can ultimately alleviate the results of necroptosis caused by OTA, we performed a flow cytometry test on CSIECs. As shown in Figure 5C, the necroptosis rate of AST group was significantly decreased (*p* < 0.05), and the necroptosis rate of OTA group was significantly increased (*p* < 0.01) compared with the CON group. Meanwhile, the necroptosis rate of the OTA + AST group was significantly decreased (*p* < 0.01) compared with the OTA group. This indicates that AST alleviated the necroptosis of OTA on CSIECs by interfering with *RIPK1/RIPK3/MLKL* signaling pathway.

### 3.6. Nec-1 Promoted the Antagonistic Effect of AST on OTA-Induced Necroptosis and Injury

**Figure 6:**
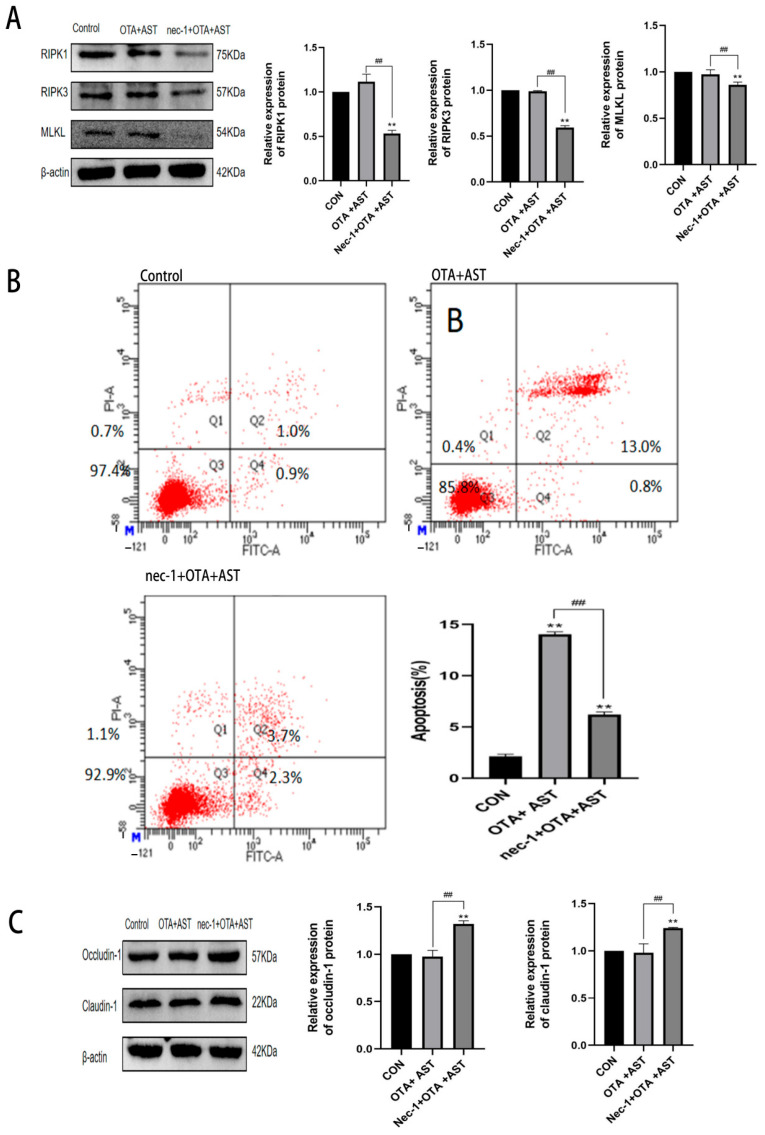
Nec-1 promoted the antagonistic effect of AST on OTA-induced necroptosis and injury. (**A**) Results of OTA, AST, and Nec-1 on key protein content of *RIPK1/RIPK3/MLKL* pathway in CSIECs (*n* = 3). (**B**) Results of OTA, AST, and Nec-1 on necroptosis of CSIECs detected by flow cytometry (*n* = 3). (**C**) Results of OTA, AST, and Nec-1 on the relative content of tight junction proteins in CSIECs (*n* = 3). Values are expressed as AVG ± SD. ** *p* < 0.01 vs. control; ## *p* < 0.01 vs. OTA group.

To further verify the effect of *RIPK1/RIPK3/MLKL* pathway on OTA-induced CSIEC injury, *RIPK1* inhibitor Nec-1 was added to the OTA + AST group cells. As shown in Figure 6A, compared with the OTA + AST group, the expression levels of RIPK1, RIPK3, and MLKL proteins in the Nec-1 + OTA + AST group were significantly decreased (*p* < 0.01).

In this regard, we performed flow cytometry to verify the necroptosis results. The necroptosis rate of the OTA + AST group and Nec-1 + OTA + AST group was significantly increased (*p* < 0.01) compared with the CON group. However, the necroptosis rate of the Nec-1 + OTA + AST group was significantly decreased (*p* < 0.01) compared with the OTA + AST group (Figure 6B). We further verified the intestinal barrier, as shown in Figure 6C. Compared with the CON group, the expression of tight junction proteins in the OTA + AST group did not change significantly, and the expression of tight junction proteins Occludin-1 and Claudin-1 in the Nec-1 + OTA + AST group was significantly increased (*p* < 0.01). This indicates that AST alleviates OTA-induced CSIEC damage and is related to necroptosis.

## 4. Discussion

OTA is mainly absorbed in the proximal third of the jejunum; even low doses of OTA can cause damage to the intestine of organisms under long-term exposure. It was reported that OTA (0.05 mg/kg, 21 d) exposure in *Hubbard chickens* induced intestinal villous blunting, epithelial erosion, and irregular crypt hyperplasia, ultimately compromising intestinal barrier integrity [39,40]. In addition, intestinal lipid peroxidation, apoptosis, oxidative stress, and mitochondrial dysfunction occurred in *White Pekin ducklings* after OTA (2 mg/kg, 21 d) intake [41]. In prior studies, animal responses were systematically assessed based on standardized experimental dosing protocols. These doses were classified into three distinct categories according to current OTA surveillance data: realistic exposure levels (<0.3 mg/kg), occasional high exposure (>0.3 mg/kg), and unrealistic exposure (>2 mg/kg) [42]. In order to reflect the excellent protective effect of AST on OTA and meet the realistic OTA conditions, we selected 1 mg/kg from within the range of 0.3–2 mg/kg OTA concentration to establish a 21-day in vivo exposure model of *white-feathered broiler chickens* in this experiment.

In this study, OTA increased the levels of pro-inflammatory factors (*IL-1β, IL-6*) and decreased the levels of anti-inflammatory factors (*IL-4*) in vivo and in vitro, while AST effectively alleviated this phenomenon. This is similar to OTA (250 μg/kg, 21 d), which increased the expression of *IL-1β*, *IL-6*, and *TNF-α* in mice through *TLR4/MyD88/NF-κB* [43]. In addition, it has been reported that OTA (5.5 μM, 24 h) upregulated the mRNA expression of *TNF-α*, *IL-1β*, *IL-6*, and *NF-kB* in IPEC-J2 [44]. At the same time, the results of SAKAI [45] showed that AST could inhibit the expression of inflammatory factors (*IL-1β*, *IL-6*, *TNF-α*, and *COX-2*) in mice and ultimately alleviate the development of colitis. We compared the OTA group and the OTA + AST group in vivo and in vitro to elucidate the effect of AST in alleviating OTA-induced chicken intestinal inflammation. The results indicate that AST successfully reversed the inflammatory injury of chicken small intestine caused by OTA.

It is worth noting that the pro-inflammatory effect of OTA is closely related to its activation of the iNOS/NO system. In this experiment, OTA exposure increased the expression of *iNOS* in the small intestine of chickens, increased the release of NO, and then weakened the expression of tight junction proteins, while AST effectively alleviated this phenomenon. This is similar to the results of ASSAR [46], where OTA (0.3 mg/kg, 2 mo) increased the expression of *iNOS* in the kidney and heart of rabbits and increased NO production, which was alleviated by *Aspergillus awamori*. It has also been reported that OTA (4 μM, 48 h) can cause serious damage to the intestinal barrier of tight junction proteins on IPEC-J2 [47]. Nitric oxide (NO) is a free radical signaling molecule, but its excessive production can increase the generation of IL-1, TNF-α, and IFN-γ while exacerbating immune responses; inducible nitric oxide synthase (iNOS) is generally not expressed under resting conditions but can be cytokine-induced and highly expressed during intestinal inflammation, which catalyzes the high-yield production of NO from L-arginine through oxidation of the terminal nitrogen in the guanidino group [48,49,50,51,52,53,54]. Other studies have highlighted the role of *iNOS* in the destruction of tight junctions, and excessive NO synthesis by *iNOS* can lead to intestinal barrier dysfunction [49]. Therefore, the toxicity mechanism of OTA is OTA-induced inflammation, which promotes the activation and production of iNOS and NO, which in turn further aggravates inflammation and continues to increase the damage caused by OTA to the small intestine of chickens. However, this situation is controlled and alleviated by AST. These results suggest that AST alleviates a series of damage caused by OTA-induced intestinal inflammation in chickens, which may be regulated by controlling necroptosis and *iNOS*, but this is still worthy of further experimental exploration.

The results show that OTA increased the mRNA and protein expression levels of *RIPK1*, *RIPK3*, and *MLKL* in vivo and in vitro. Similar to the results of XIE [55], OTA can induce necroptosis of MLE-12 cells through *RIP/MLKL* signaling pathway. In this experiment, after AST treatment, the necroptosis in vivo and in vitro caused by OTA was well alleviated or even restored to normal levels. Studies have shown that AST alleviates the apoptotic toxicity of testicular necrosis by inhibiting *RIPK1/RIPK3/MLKL* signaling in mice [56]. This is consistent with the results of this study. Nec-1 further upregulated the protein expression of Claudin-1 and Occludin and decreased the protein expression of RIPK1, RIPK3, and MLKL. At the same time, the results of flow cytometry also proved that the degree of necroptosis was weakened. These results demonstrated that AST alleviated the necroptosis of chicken intestine caused by OTA through *RIPK1/RIPK3/MLKL* signaling pathway.

We can speculate that OTA-induced intestinal injury in chickens leads to necroptosis and inflammation through *RIPK1/RIPK3/MLKL* pathway, but its adverse effects can be alleviated by AST based on the above research. This provides a theoretical basis and solution for the prevention and control of OTA toxicity in aquaculture production.

## 5. Conclusions

OTA has a toxic effect on the small intestine of chickens. AST can effectively reduce OTA-induced intestinal injury, reduce necroptosis, and inflammation of small intestinal cells by inhibiting *RIPK1/RIPK3/MLKL* signaling pathway, and increase the expression of intestinal tight junction proteins, thereby alleviating OTA-induced intestinal injury.

## Figures and Tables

**Table 1:** All specific upstream and downstream primer sequences for qRT-PCR.

Gene Name	Primer (5′–3′)	Sequence Number
β-actin	F:CCAGCCATGTATGTAGCCATCCAG R:AACACCATCACCAGAGTCCATCAC	NM_205518.2
IL-1β	F:CAGAAGAAGCCTCGCCTGGATTC R:GCCTCCGCAGCAGTTTGGTC	NM_204524.2
IL-6	F:AAATCCCTCCTCGCCAATCT R:CCCTCACGGTCTTCTCCATAAA	NM_204628.2
IL-4	F:CTTCCTCAACATGCGTCAGC R:TGAAGTAGTGTTGCCTGCTGC	NM_001007079.2
Occludin	F:CTGCTCTGCCTCATCTGCTTCTTC R:CCATCCGCCACGTTCTTCACC	NM_205128.1
Claudin-1	F:GACCAGGTGAAGAAGATGCGGATG R:CGAGCCACTCTGTTGCCATACC	NM_001013611.2
ZO-1	F:TCTTCCTCCTCCCGCTTCTTCAC R:AGAGATGGTGGTGTAGGCAGTGG	XM_040706827.2
RIPK1	F:GATCACGACTACGAACGAGATGGAC R:AACTGTAGCACCTTTGGAGCCTTG	NM_001397225.1
RIPK3	F:AACCACATCCTTGACATCCTTCGC R:CACTACAACCTGTGCTGCCTTCTC	XM_046925830.1
MLKL	F:CCATGGGTGGTTCCTCCTTC R:TGGATCTTCCGCACCTTAGC	XM_010717908.3

## Data Availability

The data presented in this research is available in this manuscript.
